# Hispano‐American Brain Bank on Neurodevelopmental Disorders: An initiative to promote brain banking, research, education, and outreach in the field of neurodevelopmental disorders

**DOI:** 10.1111/bpa.13019

**Published:** 2021-09-13

**Authors:** Brett D. Dufour, Lilia Albores‐Gallo, Jose Luna‐Muñoz, Randi Hagerman, Amaya Miquelajauregui, Efrain Buriticá, Wilmar Saldarriaga, Mar Pacheco‐Herrero, Ana Yris Silvestre‐Sosa, Carla Mazefsky, Holly Gastgeb, Julia Kofler, Manuel Casanova, Patrick R. Hof, Eric London, Paul Hagerman, Verónica Martínez‐Cerdeño

**Affiliations:** ^1^ Department of Pathology and Laboratory Medicine UC Davis School of Medicine Sacramento California USA; ^2^ Institute for Pediatric Regenerative Medicine Shriners Hospitals for Children Sacramento California USA; ^3^ Department of Genetic, Clinical, and Community Epidemiology Hospital Psiquiátrico Infantil “Dr. Juan N. Navarro” Universidad Nacional Autónoma de Mexico México City México; ^4^ National Dementia BioBank Ciencias Biológicas Facultad de Estudios Superiores Cuautitlán Universidad Nacional Autónoma de Mexico México City México; ^5^ Department of Pediatrics UC Davis School of Medicine Sacramento California USA; ^6^ MIND Institute UC Davis School of Medicine Sacramento California USA; ^7^ Institute of Neurobiology University of Puerto Rico School of Medicine San Juan Puerto Rico; ^8^ Department of Morphology Centro de Estudios Cerebrales Faculty of Health University of Valle Cali Colombia; ^9^ Universidad del valle Hospital Universitario del Valle Cali Colombia; ^10^ Neuroscience Research Laboratory Faculty of Health Sciences Pontificia Universidad Católica Madre y Maestra Santiago de los Caballeros Dominican Republic; ^11^ Hospital Santo Socorro Santo Domingo Dominican Republic; ^12^ Department of Psychiatry University of Pittsburgh School of Medicine Pittsburgh Pennsylvania USA; ^13^ Department of Pathology University of Pittsburgh School of Medicine Pittsburgh Pennsylvania USA; ^14^ Department of Biomedical Sciences School of Medicine Greenville University of South Carolina Greenville South Carolina USA; ^15^ Nash Family Department of Neuroscience Friedman Brain Institute Icahn School of Medicine at Mount Sinai New York New York USA; ^16^ New York State Institute for Basic Research Staten Island New York USA; ^17^ Department of Biochemistry University of California‐Davis Medical Center Sacramento California USA

**Keywords:** autism, brain bank, Down syndrome, Fragile X syndrome, Latinoamerica, neurodevelopmental disorders

## Abstract

Neurodevelopmental disorders (NDDs) are conditions that present with brain dysfunction due to alterations in the processes of brain development. They present with neuropsychiatric, cognitive, and motor symptoms. Autism spectrum disorder (ASD) and Fragile X syndrome (FXS) are two of the most common NDDs. Human brain tissue is a scarce resource that is obtained from postmortem donations. In the case of NDDs, specifically autism, the reduced donation rate of brains prevents researchers to investigate its pathology and fine anatomy. The Hispano‐American Brain Bank of Neurodevelopmental Disorders (Banco Hispanoamericano de **CE**rebros de trastornos del **NE**urodesarrollo) or CENE is a large‐scale brain bank for neurodevelopmental disorders in Hispano‐America and the US. CENE's objectives are to collect and distribute brains of patients with NDDS, with a focus on ASD and FXS, to perform research, promote education of future scientists, and enhance public awareness about the importance of human tissue availability for scientific research on brain function and disease. CENE has thus far established a bilingual system of nodes and teams in several American countries including California‐US, Pennsylvania‐US, México, Puerto Rico, Colombia, and Dominican Republic. CENE ensures that postmortem NDD samples used in research better match the world's genetic and ethnic diversity. CENE enables and expands NDD brain research worldwide, particularly with respect to ASD and FXS.

## Graphical abstract

CENE (Hispano – American Brain Bank of Neurodevelopmental Disorders ‐ Banco Hispanoamericano de CErebros de trastornos del NEurodesarrollo) is an organization with four goals: collecting and distributing brains of patients with ASD, FXS, and other NDDs; performing research; promoting education of future scientists; and enhancing public awareness about the importance of human tissue availability for scientific research on brain function and disease. These activities are performed in Spanish and English, in Hispano‐America and the US.

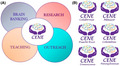

## CONFLICT OF INTEREST

LAG receives honorarium from CRIDI interviews. RH has received funding for treatment studies in FXS and Rett syndrome from Zynerba, Neuren, Ovid, and the Azrieli Foundation, and she has consulted on treatment studies for Zynerba, Ovid, and Fulcrum in the past. PJH is on the Scientific Advisory Board of NeuCyte, Inc.; he has received funding for FXS research studies from Eperium, Juvenescence‐Souvien, BioMarin, and the Azrieli Foundation. VMC consults on studies for FXS for Paxmedica. The rest of the authors declare not conflict of interest.

## AUTHOR CONTRIBUTIONS

Verónica Martínez‐Cerdeño wrote the manuscript. Brett D. Dufour contributed to the writing of the manuscript. The rest of authors revised the manuscript.

Neurodevelopmental disorders (NDDs) are conditions that present with brain dysfunction due to alterations in the processes of brain development, and that present with neuropsychiatric, cognitive, and motor symptoms. Autism spectrum disorder (ASD) and Fragile X syndrome (FXS) are among the most common NDDs. In 2016, the centers for disease control and prevention (CDC) estimated that 1 in 54 children in the US (1.85%) had autism [1]. ASD has been steadily increasing in prevalence within the US over decades (2.5‐fold increase in the US from 0.67% prevalence in 2002 [1]). FXS is the most prevalent monogenic condition that produces autism, with a prevalence of 1:5000 men and 1:4000–8000 women [2]. Other NDDs include Down syndrome, and other learning disabilities, intellectual disabilities, and conduct disorders. In the US, about 16.9% of children present with an NDD [3]. Diagnosis and treatment of these disorders is difficult. While some symptoms of NDDs resolve over time, others are permanent.

Human brain tissue is a scarce resource that is obtained from postmortem donations. Postmortem donations in cases of neurodegenerative conditions, where the donors are of advanced age, are more common and numerous brain banks for these conditions exist around the world. In the case of NDDs, specifically ASD, the reduced brain donation rate prohibits researchers from investigating the pathology and fine anatomy of these conditions. This lack of tissue is a critical shortcoming for research in America and the rest of the world. Most brain banks are located in regions largely composed of individuals with predominantly Western European heritage. Accordingly, most of the brain tissue housed at brain banks in the US and around the world, including ASD cases, come from Caucasian donors. Hispano‐America offers a great number of potential donors and a broad representation of ethnic backgrounds, but cultural, economic, and political barriers might have limited international brain‐banking collaborations. Native American, White, and Black populations inhabit Hispano‐America. The Hispano‐American Brain Bank on Neurodevelopmental Disorders (Banco Hispanoamericano de CErebros del trastornos del NEurodesarrollo – CENE) has been created to increase the number of brains available for research of NDDs and to increase representation of Hispanic and also African brains for this type of research.

## 1 CENE OBJECTIVE: TISSUE DISTRIBUTION, RESEARCH, EDUCATION, AND OUTREACH

**FIGURE 1:**
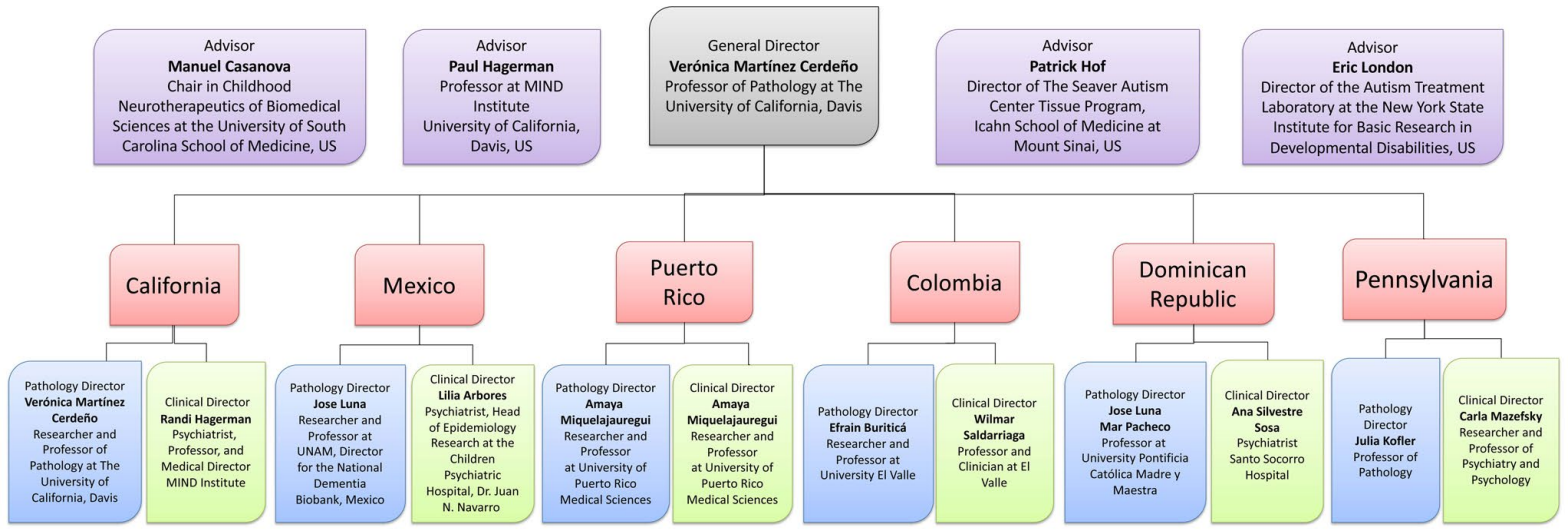
CENE has a system of nodes and teams in several American countries: United States (California, Pennsylvania) Puerto Rico, México, Colombia, and the Dominican Republic. Each node is divided in a pathology and clinical areas, and a qualified researcher and/or clinician is in charge of each of these areas. CENE has a board of advisors that are word‐wide leaders in postmortem autism research [Colour figure can be viewed at wileyonlinelibrary.com]

CENE has a system of nodes and teams in several American countries: United States (California, Pennsylvania) Puerto Rico, México, Colombia, and the Dominican Republic. This makes CENE a very valuable tool to move research on ASD to a more pluralistic and inclusive scenario. The California node emerged from the “Brain Repository in FXS and FXTAS (Fragile X‐associated Tremor and Ataxia Syndrome)” at UC Davis. The Mexico node emerged from the “Banco de Biodemencias” in Mexico. Each node is divided in a pathology and clinical areas, and a qualified researcher and/or clinician is in charge of each of these areas. The pathology directors are responsible for brain extraction, tissue preservation, and tissue distribution, while the clinical directors are in charge of localizing donors, obtaining consents for donations, verifying diagnosis, and establishing a net of psychiatrists, psychologists, and pediatricians experts in ASD and NDDs in each country. Each director has a team of collaborators in their own country. CENE has a board of advisors that are word‐wide leaders in postmortem autism research. Refer to Figure 1 for a list of node directors and advisors.

**FIGURE 2:**
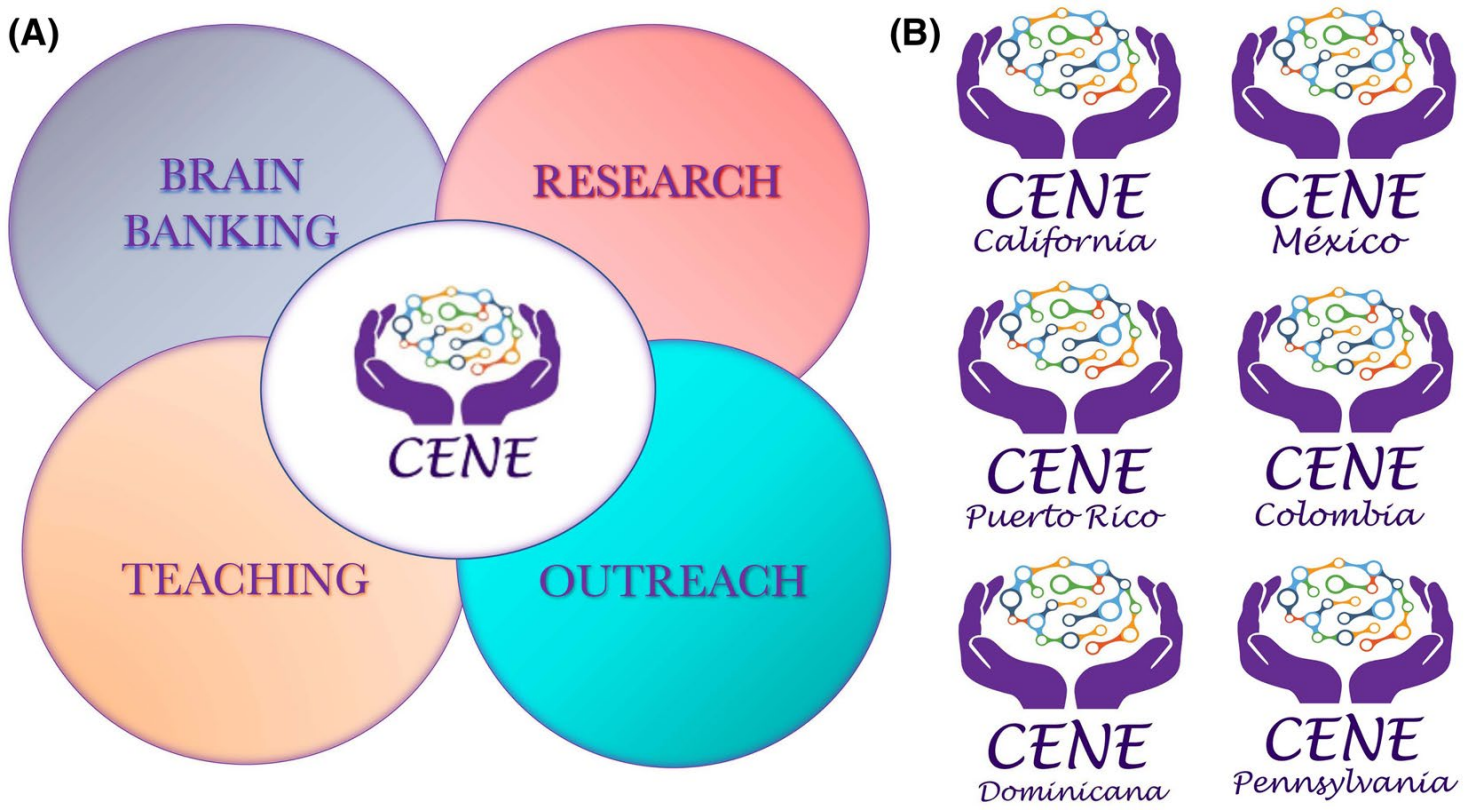
CENE is an organization with four goals: collecting and distributing brains of patients with ASD, FXS, and other NDDs; performing research; promoting young researchers education; and enhancing public awareness about the importance of human tissue availability for scientific research on brain function and disease. These activities are performed in Spanish and English, in Hispano‐America and the US [Colour figure can be viewed at wileyonlinelibrary.com]

CENE is an organization with four goals: collecting and distributing brains of patients with ASD, FXS, and other NDDs; performing research; promoting education of future scientists; and enhancing public awareness about the importance of human tissue availability for scientific research on brain function and disease (Figure 2). These activities are performed in Spanish and English, in Hispano‐America and the US.

CENE collects brains from patients with ASD and FXS, as well as brains with Down syndrome and other NDDs. Brains are from Hispanic and any other race and ethnicity donors with NDDs, as well as from neurotypical donors. ASD prevalence and brain donation rate among Hispanic people are low within Hispano‐America and the US. Brains with a suspected ASD diagnosis have their diagnosis confirmed via the Autism Diagnostic Interview‐Revised (ADI‐R). This interview is carried out with the next‐of‐kin or caretaker of the donor by a CENE clinician. An alternative Spanish‐language instrument called Criteria diagnostic interview (CRIDI) [4] is frequently used in Mexico and other parts of Hispano‐America. FXS and Down syndrome diagnoses are confirmed through genetic testing. Tissue collected and distributed by CENE so far has made possible numerous molecular, genetic, and pathology studies. More than 20 projects have been published using tissue from CENE. These studies have added a great deal of knowledge to the understanding of NDDs, particularly for FXS. An example of a project made possible by tissue from CENE resulted in the discovery by Sun et al. that unstable expansion of short tandem repeats (STRs) is an underlying mechanism for inherited NDDs, including FXS [5]. Another project carried out using tissue distributed by CENE is the work by Tran et al. who performed transcriptomic analyses that revealed that the Fragile X proteins FMRP and FXR1P interact with RNA‐editing enzymes and modulate A‐to‐I editing [6]. CENE also promotes research collaborations among nodes, with the intention of supporting the development of young researchers focused on the study of ASD and other NDDs. An example of the work carried out by the node in California, is that from Salcedo‐Arellano et al. (2021) who found intranuclear inclusions in the endothelial cells of capillaries and an increased number of cerebral microbleeds in the brains of patients with FXTAS, both of which are indicators of cerebrovascular dysfunction [7]. CENE also promotes student interchange between nodes, including trainees at the undergraduate, graduate, postdoctoral levls, and early‐career professors. Another one of CENE's goals is to organize outreach activities, which includes distributing information to patients at physician's offices and hospitals, to foundations dedicated to NDDs, through social media dedicated to NDD education (https://www.facebook.com/CENEBrainBank; https://twitter.com/CENEBrainBank), through presentations at scientific conferences directed to researchers and physicians, and at public conferences directed to patients and families.

## 2 CENE PROTOCOL FOR BRAIN OBTENTION

**FIGURE 3:**
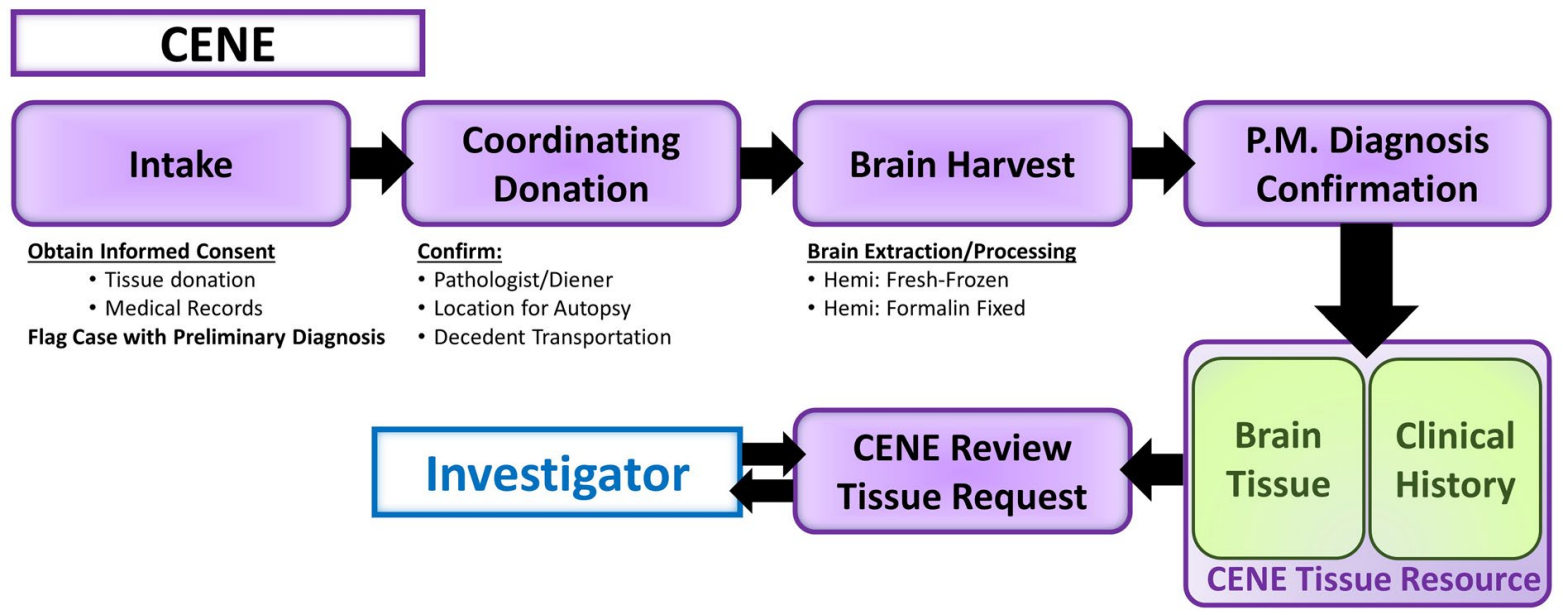
Brain donations follow a multi‐step and collaborative process that involves CENE representatives, next‐of‐kin of the donor, and pathologists in the locality of the donation [Colour figure can be viewed at wileyonlinelibrary.com]

Brain donations follow a multi‐step and collaborative process that involves CENE representatives, donor's next‐of‐kin, and pathologists in the locality of the donation (Figure 3). Upon an imminent donation, CENE is contacted by the donor's next‐of‐kin. CENE provides the family with information regarding the brain donation procedure, including a list of subject rights and researcher responsibilities. If the next‐of‐kin wishes to donate, they will sign an informed consent release, and subsequently CENE will procure a pathologist to perform the brain removal/donation and secure a location where brain procedure can take place. Consent and donation arrangements are performed preferentially before death. After brain removal, the right hemisphere is immersed in formalin while the left hemisphere is cut into a series of 1‐cm‐thick coronal slabs placed immediately on dry ice to freeze. If postmortem interval is more than 24 h, the whole brain is immersed in formalin. Tissue is stored in formalin until used. Fixed tissue is used for generating a neuropathology report. For FXS cases, we determine the CCG repeat number for FXS cases using PCR. Tissue collected by CENE is shared with any researcher around the world who demonstrates an institutional affiliation, professional degree, basic expertise in the use of postmortem tissue for research and has legitimate research objectives. Our goal is to distribute tissue free of cost (except for shipping), as funding allows. Researchers are asked for a document briefly describing the project that requires the use of the requested human tissue. This document is reviewed by the tissue distribution committee formed by members of the nodes, and if approved (it is the case for the majority of applications), the researchers are asked to specify the type and quantity of tissue required. The type of tissue provided includes fresh frozen and formalin‐fixed tissue. Fresh frozen tissue for genetic and protein analysis is provided in small amounts (100 mg) isolated from the anatomical requested, fixed tissue for immunohistochemical experiments is provided in precut slide‐mounted sections. Sections are generally 14‐µm‐thick (cryostat sections) or 50‐µm‐thick (freezing microtome sections), after cryoprotection of the specimen. Paraffin sections are provided after special request. However, if the researcher needs intact tissue blocks for any methodological reason, fixed tissue is instead provided in this manner. If the slices are already cut, the whole process of tissue request and distribution should take less than 1–2 weeks. If the slices need to be cut, the process will take longer.

## 3 CENE SUPPORTING INSTITUTIONS AND ORGANIZATIONS

CENE supporting institutions and organizations include the Institute for Medical Investigation of Neurodevelopmental Disorders (MIND), University of California, Davis, USA; the School of Advanced Studies (Facultad de estudios superiores) Cuautitlán, UNAM, Mexico; the Católica Madre y Maestra University (Pontificia Universidad Católica Madre y Maestra) Dominican Republic; The Brain Studies Center (Centro de Estudios Cerebrales), Universidad del Valle, Colombia; the National Dementia BioBank of Mexico (BioBanco Nacional de Demencias de México), Mexico; the Latino‐American Net of Brain Banks (Red Latino‐Americana de bancos de cerebros); the Latino‐American Net of Professional on Neurodevelopment (Red Latino‐Americana de profesionales del Neurodesarrollo), PROCEDA (Profesionales certificados en detección y diagnóstico de Autismo), México; among others.

To learn more about CENE go to our website:

https://health.ucdavis.edu/mindinstitute/research/cene‐brain‐bank/cene‐about.html (For Spanish version: https://health.ucdavis.edu/mindinstitute/research/cene‐brain‐bank/index‐span.html) or to https://ventricular.org/NEWCENE/en/home/.

Contact us by e‐mail: vmartinezcerdeno@ucdavis.edu or cenebrainbank@ventricular.org.

## Data Availability

Data will be provided upon request to the corresponding author.
